# Supplementary material for: Pharmacological vs. Non-Pharmacological Treatment in the Management of Relative Energy Deficiency in Sport (REDs): A Systematic Review and Meta-Analysis
Source: Sports (Basel). 2025 Dec 15;13(12):453. doi: 10.3390/sports13120453 (PMC12737033; doi:10.3390/sports13120453)
Supplement: Supplementary file 1 [file sports-13-00453-s001.zip › sports-3994941-supplementary.pdf]

Supplementary file

1. PRISMA checklist completed (S1)
2. Critical appraisal assessment (S2)
3. Certainty Assessment (S3)

PRISMA checklist (adapted from Page et al [1])

Section and Topic	Item #	Checklist item	Location where item is reported
**TITLE**			
Title	1	Identify the report as a systematic review.	1
**ABSTRACT**			
Abstract	2	See the PRISMA 2020 for Abstracts checklist.	1
**INTRODUCTION**			
Rationale	3	Describe the rationale for the review in the context of existing knowledge.	2-3
Objectives	4	Provide an explicit statement of the objective(s) or question(s) the review addresses.	3
**METHODS**			
Eligibility criteria	5	Specify the inclusion and exclusion criteria for the review and how studies were grouped for the syntheses.	3-4
Information sources	6	Specify all databases, registers, websites, organisations, reference lists and other sources searched or consulted to identify studies. Specify the date when each source was last searched or consulted.	4
Search strategy	7	Present the full search strategies for all databases, registers and websites, including any filters and limits used.	4
Selection process	8	Specify the methods used to decide whether a study met the inclusion criteria of the review, including how many reviewers screened each record and each report retrieved, whether they worked independently, and if applicable, details of automation tools used in the process.	5
Data collection process	9	Specify the methods used to collect data from reports, including how many reviewers collected data from each report, whether they worked independently, any processes for obtaining or confirming data from study investigators, and if applicable, details of automation tools used in the process.	4
Data items	10a	List and define all outcomes for which data were sought. Specify whether all results that were compatible with each outcome domain in each study were sought (e.g. for all measures, time points, analyses), and if not, the methods used to decide which results to collect.	5
	10b	List and define all other variables for which data were sought (e.g. participant and intervention characteristics, funding sources). Describe any assumptions made about any missing or unclear information.	5
Study risk of bias assessment	11	Specify the methods used to assess risk of bias in the included studies, including details of the tool(s) used, how many reviewers assessed each study and whether they worked independently, and if applicable, details of automation tools used in the process.	5
Effect measures	12	Specify for each outcome the effect measure(s) (e.g. risk ratio, mean difference) used in the synthesis or presentation of results.	5
Synthesis methods	13a	Describe the processes used to decide which studies were eligible for each synthesis (e.g. tabulating the study intervention characteristics and comparing against the planned groups for each synthesis (item #5)).	5
	13b	Describe any methods required to prepare the data for presentation or synthesis, such as handling of missing summary statistics, or data conversions.	5
	13c	Describe any methods used to tabulate or visually display results of individual studies and syntheses.	5
	13d	Describe any methods used to synthesize results and provide a rationale for the choice(s). If meta-analysis was performed, describe the model(s), method(s) to identify the presence and extent of statistical heterogeneity, and software package(s) used.	5
	13e	Describe any methods used to explore possible causes of heterogeneity among study results (e.g. subgroup analysis, meta-regression).	5
	13f	Describe any sensitivity analyses conducted to assess robustness of the synthesized results.	n/a
Reporting bias assessment	14	Describe any methods used to assess risk of bias due to missing results in a synthesis (arising from reporting biases).	5
Certainty assessment	15	Describe any methods used to assess certainty (or confidence) in the body of evidence for an outcome.	5
**RESULTS**			
Study selection	16a	Describe the results of the search and selection process, from the number of records identified in the search to the number of studies included in the review, ideally using a flow diagram.	6
	16b	Cite studies that might appear to meet the inclusion criteria, but which were excluded, and explain why they	6

Section and Topic	Item #	Checklist item	Location where item is reported
		were excluded.	
Study characteristics	17	Cite each included study and present its characteristics.	6-20
Risk of bias in studies	18	Present assessments of risk of bias for each included study.	Supplementary file (incl. in certainty assessment)
Results of individual studies	19	For all outcomes, present, for each study: (a) summary statistics for each group (where appropriate) and (b) an effect estimate and its precision (e.g. confidence/credible interval), ideally using structured tables or plots.	20-22
Results of syntheses	20a	For each synthesis, briefly summarise the characteristics and risk of bias among contributing studies.	20-22
	20b	Present results of all statistical syntheses conducted. If meta-analysis was done, present for each the summary estimate and its precision (e.g. confidence/credible interval) and measures of statistical heterogeneity. If comparing groups, describe the direction of the effect.	20-22
	20c	Present results of all investigations of possible causes of heterogeneity among study results.	6-22
	20d	Present results of all sensitivity analyses conducted to assess the robustness of the synthesized results.	n/a
Reporting biases	21	Present assessments of risk of bias due to missing results (arising from reporting biases) for each synthesis assessed.	Supplementary file (incl. in certainty assessment)
Certainty of evidence	22	Present assessments of certainty (or confidence) in the body of evidence for each outcome assessed.	20-22
**DISCUSSION**			
Discussion	23a	Provide a general interpretation of the results in the context of other evidence.	22-26
	23b	Discuss any limitations of the evidence included in the review.	26
	23c	Discuss any limitations of the review processes used.	26
	23d	Discuss implications of the results for practice, policy, and future research.	26-27
**OTHER INFORMATION**			
Registration and protocol	24a	Provide registration information for the review, including register name and registration number, or state that the review was not registered.	3
	24b	Indicate where the review protocol can be accessed, or state that a protocol was not prepared.	3
	24c	Describe and explain any amendments to information provided at registration or in the protocol.	3
Support	25	Describe sources of financial or non-financial support for the review, and the role of the funders or sponsors in the review.	27
Competing interests	26	Declare any competing interests of review authors.	27
Availability of data, code and other materials	27	Report which of the following are publicly available and where they can be found: template data collection forms; data extracted from included studies; data used for all analyses; analytic code; any other materials used in the review.	27

References

1. Page MJ, McKenzie JE, Bossuyt PM, Boutron I, Hoffmann TC, Mulrow CD, et al. The PRISMA 2020 statement: an updated guideline for reporting systematic reviews. BMJ 2021;372:n71. doi: 10.1136/bmj.n71. This work is licensed under CC BY 4.0. To view a copy of this license, visit https://creativecommons.org/licenses/by/4.0/

Critical Appraisal

The following risk of bias tools were used depending on study design: ROB 2 (for Randomised Control trials) (RCTs, n= 7/19, 37%). See Table 1 for summary of the randomised studies. ROBINS-I V2 (for non-randomised interventional studies, n= 11, 58%), and the JBI checklist (for case reports, n=1 , 5%). See Table 2 for a summary of the quality of non randomised studies.

Overall, RCTs demonstrated low (n= 3/7, 43%) to moderate (n= 4/7, 57%) risk of bias, with most (n= 5/7, 71%) studies showing low risk across all key domains. However, two RCTs [1,2] had "some concerns" particularly in randomisation and outcome reporting. In contrast, all non-randomised studies were rated as having serious risk due to the influence of confounding variables (e.g. baseline differences of energy availability, variation in training load, duration of menstrual dysfunction, age). Additional concerns were noted in non-RCTs in domains such as intervention classification (serious risk (n= 1/11, 9%), moderate risk (n= 10/11, 91%)), outcome measurement (n= 10/11, 91%), and missing data (n= 6/11, 55%), typically rated at moderate risk. The single case report by Mallinson et al. [3] was methodologically sound across most JBI criteria but lacked reporting on adverse or unanticipated events. See Table 3 for the critical appraisal of the case study.

Table 1 Critical Appraisal of Randomised Studies

**ROB 2**						
	bias arising from the randomisation process	bias due to deviations from intended interventions	bias due to missing outcome data	bias in measurem ent of the outcome	bias in selection of reported results	overal l ranki ng
Randomised controlled trial of the effects of increased energy intake on menstrual recovery in exercising women with menstrual disturbances: the 'REFUEL' study. De Souza et al (2021)[4]	Low	Low	Moderate	Low	Low	low
Treatment of reduced bone mineral density in athletic amenorrhea: a pilot study Gibson et al (1999) [5]	Low	Low-Moderate	Moderate	Low	Low	mode rate
Persistent osteopenia in ballet dancers with amenorrhea and delayed menarche despite hormone therapy: a longitudinal study Warren et al (2003) [6]	Low	Low	Some concerns	Low	Low	low
Neuroendocrine recovery initiated by cognitive behavioral therapy in women with functional hypothalamic amenorrhea: a randomized, controlled trial Michopoulos et al (2013) [7]	Some concerns	Some concerns	Low	Low	Some concerns	mode rate

Oestrogen replacement improves bone mineral density in oligo-amenorrhoeic athletes: a randomised clinical trial Ackerman et al. (2020) [8]	Low	Some concerns	Low	Low	Some concerns	mode rate
Bone mineral density in response to increased energy intake in exercising women with oligomenorrhea/amenorrhea: the REFUEL randomized controlled trial De Souza et al. (2022) [9]	Low	Some concerns	Low	Low	Low	low
The effect of hormone therapy on bone mineral density and cardiovascular factors among Iranian female athletes with amenorrhea/oligomenorrhea: A randomised clinical trial Dadgostar et al (2018) [10]	Some concerns	Some concerns	Low	Low	Some concerns	mode rate

Table 2 Critical Appraisal of non-randomised studies

ROBINS I V2							
	risk of bias due to confounding	risk of bias in classification of intervention	risk of bias in selection into the study	risk of bias due to deviations from intended intervention	risk of bias due to missing data	risk of bias arising from measurement of the outcome	risk of bias in selection of the reported results
Energy and Nutrient Status of Amenorrheic Athletes Participating in a Diet and Exercise Training Intervention Programme Kopp-Woodroffe et al. (1999) [11]	serious risk	moderate risk	low risk	moderate risk	low risk	moderate risk	moderate risk
Participant evaluations of the FUEL intervention designed for female endurance athletes at risk of REDs: A mixed methods approach. Solstad et al. (2025) [12]	serious risk	moderate risk	low risk	low risk	moderate risk	moderate risk	moderate risk
Healthy Runner Project: a 7-year, multisite nutrition education intervention to reduce bone stress injury incidence in collegiate distance runners Fredericson et al (2023) [13]	serious risk	moderate risk	low risk	moderate risk	low risk	low risk	moderate risk
Effects of a 16-week digital intervention on sports nutrition knowledge and behavior in female endurance athletes with risk of relative energy deficiency in sport (REDs) Fahrenholtz et al (2023) [14]	serious risk	moderate risk	low risk	low risk	moderate risk	moderate risk	low risk
Dietary intervention restored menses in female athletes with exercise-associated menstrual dysfunction with limited impact on bone and muscle health Cialdella-Kam et al (2014) [15]	serious risk	serious risk	low risk	moderate risk	low risk	moderate risk	moderate risk

Active women before/after an intervention designed to restore menstrual function: resting metabolic rate and comparison of four methods to quantify energy expenditure and energy availability Guebels et al (2014) [16]	serious risk	moderate risk	low risk	moderate risk	low risk	moderate risk	moderate risk
Nine–month nutritional intervention improves restoration of menses in young female athletes and ballet dancers Lagowska et al (2014) [17]	serious risk	moderate risk	low risk	moderate risk	moderate risk	moderate risk	moderate risk
Effects of dietary intervention in young female athletes with menstrual disorders Łagowska et al (2014) [18]	serious risk	moderate risk	low risk	moderate risk	moderate risk	moderate risk	moderate risk
Treatment of athletic amenorrhea with a diet and training intervention program Dueck et al., (1996) [19]	serious risk	moderate risk	low risk	moderate risk	low risk	moderate risk	moderate risk
Restoration of menses with nonpharmacologic therapy in college athletes with menstrual disturbances: a 5-year retrospective study Arendns et al (2012) [20]	serious risk	moderate risk	moderate risk	serious risk	serious risk	moderate risk	moderate risk
Relative Energy Deficiency in Sport—Multidisciplinary Treatment in Clinical Practice Meyer et al (2025) [21]	serious risk	moderate risk	moderate risk	serious risk	moderate-serious risk	moderate risk	moderate risk

Table 3 Critical Appraisal of a case report study

JBI								
	Were patient's demographic characteristics clearly described?	Was the patient's history clearly described and presented as a timeline?	Was the current clinical condition of the patient on presentation clearly described?	Were diagnostic tests or assessment methods and the results clearly described?	Was the intervention(s) or treatment procedure(s) clearly described?	Was the post-intervention clinical condition clearly described?	Were adverse events (harms) or unanticipated events identified and described?	Does the case report provide takeaway lessons?
A case report of recovery of menstrual function following a nutritional intervention in two exercising women with amenorrhea of varying duration Mallinson et al. (2013)	yes	yes	yes	yes	yes	yes	no	yes

References

1. Michopoulos, V., Mancini, F., Loucks, T. L., & Berga, S. L. (2013). Neuroendocrine recovery initiated by cognitive behavioral therapy in women with functional hypothalamic amenorrhea: A randomized, controlled trial. Fertility and Sterility, 99(7), 2084–2091.e1. https://doi.org/10.1016/j.fertnstert.2013.02.036

2. Dadgostar, H., Soleimany, G., Movaseghi, S., Dadgostar, E., & Lotfian, S. (2018). The effect of hormone therapy on bone mineral density and cardiovascular factors among Iranian female athletes with amenorrhea/oligomenorrhea: A randomized clinical trial. Medical Journal of the Islamic Republic of Iran, 32, 27. https://doi.org/10.14196/mjiri.32.27

3. Mallinson, R. J., Williams, N. I., Olmsted, M. P., Scheid, J. L., Riddle, E. S., & De Souza, M. J. (2013). A case report of recovery of menstrual function following a nutritional intervention in two exercising women with amenorrhea of varying duration. Journal of the International Society of Sports Nutrition, 10(1), 34. https://doi.org/10.1186/1550-2783-10-34

4. De Souza, M. J., Mallinson, R. J., Strock, N. C. A., Koltun, K. J., Olmsted, M. P., Ricker, E. A., Scheid, J. L., Allaway, H. C., Mallinson, D. J., Kuruppumullage Don, P., et al. (2021). Randomised controlled trial of the effects of increased energy intake on menstrual recovery in exercising women with menstrual disturbances: The 'REFUEL' study. Human Reproduction, 36(8), 2285–2297. https://doi.org/10.1093/humrep/deab149

5. Gibson, J.H.; Mitchell, A.; Reeve, J.; Harries, M.G. Treatment of reduced bone mineral density in athletic amenorrhea: A pilot study. Osteoporos. Int. 1999, 10, 284–289. https://doi.org/10.1007/s001980050228

6. Warren, M. P., Brooks-Gunn, J., Fox, R. P., Holderness, C. C., Hyle, E. P., Hamilton, W. G., & Hamilton, L. (2003). Persistent osteopenia in ballet dancers with amenorrhea and delayed menarche despite hormone therapy: A longitudinal study. Fertility and Sterility, 80(2), 398–404. https://doi.org/10.1016/S0015-0282(03)00660-5

7. Michopoulos, V.; Mancini, F.; Loucks, T.L.; Berga, S.L. Neuroendocrine recovery initiated by cognitive behavioral therapy in women with functional hypothalamic amenorrhea: A randomized, controlled trial. Fertil. Steril. 2013, 99, 2084–2091.e1. https://doi.org/10.1016/j.fertnstert.2013.02.036.

8. Ackerman, K. E., Singhal, V., Slattery, M., Eddy, K. T., Bouxsein, M. L., Lee, H., Klibanski, A., & Misra, M. (2020). Effects of estrogen replacement on bone geometry and microarchitecture in adolescent and young adult oligoamenorrheic athletes: A randomized trial. Journal of Bone and Mineral Research, 35(2), 248–260. https://doi.org/10.1002/jbmr.3887

9. De Souza, M. J., Ricker, E. A., Mallinson, R. J., Allaway, H. C. M., Koltun, K. J., Strock, N. C. A., Gibbs, J. C., Kuruppumullage Don, P., & Williams, N. I. (2022). Bone mineral density in response to increased energy intake in exercising women with oligomenorrhea/amenorrhea: The REFUEL randomized controlled trial. American Journal of Clinical Nutrition, 115(6), 1457–1472. https://doi.org/10.1093/ajcn/nqac044

10. Dadgostar, H.; Soleimany, G.; Movaseghi, S.; Dadgostar, E.; Lotfian, S. The effect of hormone therapy on bone mineral density and cardiovascular factors among Iranian female athletes with amenorrhea/oligomenorrhea: A randomized clinical trial. Med J. Islam. Repub. Iran 2018, 32, 27. https://doi.org/10.14196/mjiri.32.27.

11. Kopp-Woodroffe, S. A., Manore, M. M., Dueck, C. A., Skinner, J. S., & Matt, K. S. (1999). Energy and nutrient status of amenorrheic athletes participating in a diet and exercise training intervention program. International Journal of Sport Nutrition, 9(1), 70–88. https://doi.org/10.1123/ijsn.9.1.70

12. Solstad, B.E.; Fahrenholtz, I.L.; Melin, A.; Garthe, I.; Torstveit, M.K. Participant evaluations of the FUEL intervention designed for female endurance athletes at risk of REDs. A mixed methods approach. Sports Psychiatry 2025, 4, 51–70. https://doi.org/10.1024/2674-0052/a000107.

13. Fredericson, M.; Roche, M.; Barrack, M.T.; Tenforde, A.; Sainani, K.; Kraus, E.; Kussman, A.; Miller Olson, E.; Kim, B.Y.; Fahy, K.; et al. Healthy Runner Project: A 7-year, multisite nutrition education intervention to reduce bone stress injury incidence in collegiate distance runners. BMJ Open Sport Exerc. Med. 2023, 9, e001545. https://doi.org/10.1136/bmjsem-2023-001545.

14. Fahrenholtz, I.L.; Melin, A.K.; Garthe, I.; Hollekim-Strand, S.M.; Ivarsson, A.; Koehler, K.; Logue, D.; Lundström, P.; Madigan, S.; Wasserfurth, P.; et al. Effects of a 16-Week Digital Intervention on Sports Nutrition Knowledge and Behavior in

Female Endurance Athletes with Risk of Relative Energy Deficiency in Sport (REDs). Nutrients 2023, 15, 1082. https://doi.org/10.3390/nu15051082.

15. Cialdella-Kam, L.; Guebels, C.P.; Maddalozzo, G.F.; Manore, M.M. Dietary intervention restored menses in female athletes with exercise-associated menstrual dysfunction with limited impact on bone and muscle health. Nutrients 2014, 6, 3018–3039. https://doi.org/10.3390/nu6083018.

16. Guebels, C. P., Kam, L. C., Maddalozzo, G. F., & Manore, M. M. (2014). Active women before/after an intervention designed to restore menstrual function: Resting metabolic rate and comparison of four methods to quantify energy expenditure and energy availability. International Journal of Sport Nutrition and Exercise Metabolism, 24(1), 37–46. https://doi.org/10.1123/ijsnem.2012-0165

17. Lagowska, K.; Kapczuk, K.; Jeszka, J. Nine-month nutritional intervention improves restoration of menses in young female athletes and ballet dancers. J. Int. Soc. Sports Nutr. 2014, 11, 52. https://doi.org/10.1186/s12970-014-0052-9.

18. Lagowska, K.; Kapczuk, K.; Friebe, Z.; Bajerska, J. Effects of dietary intervention in young female athletes with menstrual disorders. J. Int. Soc. Sports Nutr. 2014, 11, 21. https://doi.org/10.1186/1550-2783-11-21.

19. Dueck, C. A., Matt, K. S., Manore, M. M., & Skinner, J. S. (1996). Treatment of athletic amenorrhea with a diet and training intervention program. International Journal of Sport Nutrition, 6(1), 24–40. https://doi.org/10.1123/ijsn.6.1.24

20. Arends, J. C., Cheung, M. Y. C., Barrack, M. T., & Nattiv, A. (2012). Restoration of menses with nonpharmacologic therapy in college athletes with menstrual disturbances: A 5-year retrospective study. International Journal of Sport Nutrition and Exercise Metabolism, 22(2), 98–108. https://doi.org/10.1123/ijsnem.22.2.98

21. Meyer, A., Haigis, D., Klos, B., Zipfel, S., Resmark, G., Rall, K., Dreser, K., Hagmann, D., Nieß, A., Kopp, C., et al. (2025). Relative energy deficiency in sport—Multidisciplinary treatment in clinical practice. Nutrients, 17(2), 228. https://doi.org/10.3390/nu17020228

22. Mallinson, R.J.; Williams, N.I.; Olmsted, M.P.; Scheid, J.L.; Riddle, E.S.; De Souza, M.J. A case report of recovery of menstrual function following a nutritional intervention in two exercising women with amenorrhea of varying duration. J. Int. Soc. Sports Nutr. 2013, 10, 34. https://doi.org/10.1186/1550-2783-10-34.

Certainty Assessment Results

See table 4 for the Certainty Assessment Results.

Table 4 Certainty assessment results.

	**Menstrual function recovery**			**Energy availability**			**Body composition**			**Biomarkers**	
	GRADE assessment of certainty	*Reasons for downgrade (-) or upgrade (+)*		*GRADE assessment of certainty*	*Reasons for downgrade or upgrade*		*GRADE assessment of certainty*	*Reasons for downgrade or upgrade*		*GRADE assessment of certainty*	*Reasons for downgrade or upgrade*
Kopp-Woodroffe et al. (1999) [1]				Low	risk of bias (-1), imprecision (-1), publication bias (-1), large effect size (+1)					Low	risk of bias (-1), imprecision (-1), publication bias (-1), moderate effect size (+1)
Mallinson et al. (2013) [2]	Very low	risk of bias(-1), inconsistency (-1), imprecision (-1), publication bias (-1)					Low	risk of bias(-1), imprecision (-1), publication bias (-1), very large effect size (+1)		Low	risk of bias (-1), imprecision (-1), publication bias (-1), large effect size (+1)
De Souza et al (2021) [3]	High	Imprecision (-1), large effect (+1), dose-reponse (+1)					Very high	large effect size (+1)		Very high	
Meyer et al (2025) [4]	Low	risk of bias (-1), indirectness (-1), imprecision (-1), publication bias (-1), very large effect size (+1)					Low	risk of bias(-1), imprecision (-1), publication bias (-1), large effect size (+1)			
Cialdella-Kam et al (2014) [5]	Low	risk of bias (-1), imprecision (-1), publication bias (-1), large effect size (+1)		Low	risk of bias (-1), imprecision (-1), publication bias (-1), moderate effect size (+1)					Low	risk of bias (-1), imprecision (-1), publication bias (-1), moderate effect size (+1)

Guebels et al (2014) [6]				Low	risk of bias (-1), imprecision (-1), publication bias (-1), large effect size (+1)						
Lagowska et al (2014) [7]	Low	risk of bias (-1), imprecision (-1), publication bias (-1), large effect size (+1)		Low	risk of bias (-1), imprecision (-1), publication bias (-1), moderate effect size (+1)					Low	risk of bias (-1), imprecision (-1), publication bias (-1), moderate (+1)
Gibson et al (1999) [8]										Very high	moderate effect size (+1)
Warren et al (2003) [9]				L ow	risk of bias (-1), imprecision (-1), publication bias (-1), large effect size (+1)		Low	risk of bias(-1), imprecision (-1), publication bias (-1), moderate effect size (+1)		Low	risk of bias (-1), imprecision (-1), publication bias (-1), moderate effect (+1)
Michopoulos et al (2013) [10]	High						Very high	moderate effect size (+1)		Very high	moderate effect size (+1)
Łagowska et al (2014) [11]	Low	risk of bias (-1), imprecision (-1), publication bias (-1), large effect size (+1)					Low	risk of bias(-1), imprecision (-1), publication bias (-1), moderate effect size (+1)		Low	risk of bias (-1), imprecision (-1), publication bias (-1), moderate effect size (+1)
Ackerman et al. (2019) [12]	Low	risk of bias (-1), imprecision (-1), publication bias (-1), very large effect size (+1)					Low	risk of bias(-1), imprecision (-1), publication bias (-1), moderate effect size (+1)			

**Author**	**Bone Mineral Density (BMD) (via DXA scan)**			**Hormonal profiles**			**Menstrual function recovery**			**Bone turnover markers**	
	GRADE assessment of certainty	Reasons for downgrade (-) or upgrade (+)		GRADE assessment of certainty	Reasons for downgrade (-) or upgrade (+)		GRADE assessment of certainty	Reasons for downgrade (-) or upgrade (+)		GRADE assessment of certainty	Reasons for downgrade (-) or upgrade (+)
De Souza et al. (2022) [13]	Low	risk of bias (-1), imprecision (-1), publication bias (-1), large effect size (+1)		Low	risk of bias (-1), imprecision (-1), publication bias (-1), large effect size (+1)		Low	risk of bias (-1), imprecision (-1), publication bias (-1), large effect size (+1)			
Dueck et al., (1996) [14]	Very low	risk of bias (-1), imprecision (-1), publication bias (-1)		Very low	risk of bias (-1), imprecision (-1), publication bias (-1)		Very low	risk of bias (-1), imprecision (-1), publication bias (-1)			
Arends et al (2012) [15]	Very high	large effect size (+1)		Very high	moderate effect size (+1)		High			High	
Dadgostar et al (2018)	High	large effect size (+1)		High	moderate effect size (+1)		High			High	

References

1. Kopp-Woodroffe, S. A., Manore, M. M., Dueck, C. A., Skinner, J. S., & Matt, K. S. (1999). Energy and nutrient status of amenorrheic athletes participating in a diet and exercise training intervention program. International Journal of Sport Nutrition, 9(1), 70–88. https://doi.org/10.1123/ijsn.9.1.70
2. Mallinson, R. J., Williams, N. I., Olmsted, M. P., Scheid, J. L., Riddle, E. S., & De Souza, M. J. (2013). A case report of recovery of menstrual function following a nutritional intervention in two exercising women with amenorrhea of varying duration. Journal of the International Society of Sports Nutrition, 10(1), 34. https://doi.org/10.1186/1550-2783-10-34

3. De Souza, M. J., Mallinson, R. J., Strock, N. C. A., Koltun, K. J., Olmsted, M. P., Ricker, E. A., Scheid, J. L., Allaway, H. C., Mallinson, D. J., Kuruppumullage Don, P., et al. (2021). Randomised controlled trial of the effects of increased energy intake on menstrual recovery in exercising women with menstrual disturbances: The 'REFUEL' study. Human Reproduction, 36(8), 2285–2297. https://doi.org/10.1093/humrep/deab149
4. Meyer, A., Haigis, D., Klos, B., Zipfel, S., Resmark, G., Rall, K., Dreser, K., Hagmann, D., Nieß, A., Kopp, C., et al. (2025). Relative energy deficiency in sport—Multidisciplinary treatment in clinical practice. Nutrients, 17(2), 228. https://doi.org/10.3390/nu17020228
5. Cialdella-Kam, L.; Guebels, C.P.; Maddalozzo, G.F.; Manore, M.M. Dietary intervention restored menses in female athletes with exercise-associated menstrual dysfunction with limited impact on bone and muscle health. Nutrients 2014, 6, 3018–3039. https://doi.org/10.3390/nu6083018.
6. Guebels, C. P., Kam, L. C., Maddalozzo, G. F., & Manore, M. M. (2014). Active women before/after an intervention designed to restore menstrual function: Resting metabolic rate and comparison of four methods to quantify energy expenditure and energy availability. International Journal of Sport Nutrition and Exercise Metabolism, 24(1), 37–46. https://doi.org/10.1123/ijsnem.2012-0165
7. Lagowska, K.; Kapczuk, K.; Jeszka, J. Nine-month nutritional intervention improves restoration of menses in young female athletes and ballet dancers. J. Int. Soc. Sports Nutr. 2014, 11, 52. https://doi.org/10.1186/s12970-014-0052-9.
8. Gibson, J.H.; Mitchell, A.; Reeve, J.; Harries, M.G. Treatment of reduced bone mineral density in athletic amenorrhea: A pilot study. Osteoporos. Int. 1999, 10, 284–289. https://doi.org/10.1007/s001980050228
9. Warren, M. P., Brooks-Gunn, J., Fox, R. P., Holderness, C. C., Hyle, E. P., Hamilton, W. G., & Hamilton, L. (2003). Persistent osteopenia in ballet dancers with amenorrhea and delayed menarche despite hormone therapy: A longitudinal study. Fertility and Sterility, 80(2), 398–404. https://doi.org/10.1016/S0015-0282(03)00660-5
10. Michopoulos, V.; Mancini, F.; Loucks, T.L.; Berga, S.L. Neuroendocrine recovery initiated by cognitive behavioral therapy in women with functional hypothalamic amenorrhea: A randomized, controlled trial. Fertil. Steril. 2013, 99, 2084–2091.e1. https://doi.org/10.1016/j.fertnstert.2013.02.036.
11. Lagowska, K.; Kapczuk, K.; Friebe, Z.; Bajerska, J. Effects of dietary intervention in young female athletes with menstrual disorders. J. Int. Soc. Sports Nutr. 2014, 11, 21. https://doi.org/10.1186/1550-2783-11-21.
12. Ackerman, K. E., Singhal, V., Slattery, M., Eddy, K. T., Bouxsein, M. L., Lee, H., Klibanski, A., & Misra, M. (2020). Effects of estrogen replacement on bone geometry and microarchitecture in adolescent and young adult oligoamenorrheic athletes: A randomized trial. Journal of Bone and Mineral Research, 35(2), 248–260. https://doi.org/10.1002/jbmr.3887
13. De Souza, M. J., Ricker, E. A., Mallinson, R. J., Allaway, H. C. M., Koltun, K. J., Strock, N. C. A., Gibbs, J. C., Kuruppumullage Don, P., & Williams, N. I. (2022). Bone mineral density in response to increased energy intake in exercising women with oligomenorrhea/amenorrhea: The REFUEL randomized controlled trial. American Journal of Clinical Nutrition, 115(6), 1457–1472. https://doi.org/10.1093/ajcn/nqac044
14. Dueck, C. A., Matt, K. S., Manore, M. M., & Skinner, J. S. (1996). Treatment of athletic amenorrhea with a diet and training intervention program. International Journal of Sport Nutrition, 6(1), 24–40. https://doi.org/10.1123/ijsn.6.1.24
15. Arends, J. C., Cheung, M. Y. C., Barrack, M. T., & Nattiv, A. (2012). Restoration of menses with nonpharmacologic therapy in college athletes with menstrual disturbances: A 5-year

retrospective study. International Journal of Sport Nutrition and Exercise Metabolism, 22(2), 98–108. https://doi.org/10.1123/ijsnem.22.2.98

16. Dadgostar, H., Soleimany, G., Movaseghi, S., Dadgostar, E., & Lotfian, S. (2018). The effect of hormone therapy on bone mineral density and cardiovascular factors among Iranian female athletes with amenorrhea/oligomenorrhea: A randomized clinical trial. Medical Journal of the Islamic Republic of Iran, 32, 27. https://doi.org/10.14196/mjiri.32.27
